# Alzheimer disease stages identification based on correlation transfer function system using resting-state functional magnetic resonance imaging

**DOI:** 10.1371/journal.pone.0264710

**Published:** 2022-04-12

**Authors:** Doaa Mousa, Nourhan Zayed, Inas A. Yassine

**Affiliations:** 1 Computers and Systems Department, Electronics Research Institute, Giza, Egypt; 2 Systems and Biomedical Engineering Department, Cairo University, Giza, Egypt; Nathan S Kline Institute, UNITED STATES

## Abstract

Alzheimer’s disease (AD) affects the quality of life as it causes; memory loss, difficulty in thinking, learning, and performing familiar tasks. Resting-state functional magnetic resonance imaging (rs-fMRI) has been widely used to investigate and analyze different brain regions for AD identification. This study investigates the effectiveness of using correlated transfer function (CorrTF) as a new biomarker to extract the essential features from rs-fMRI, along with support vector machine (SVM) ordered hierarchically, in order to distinguish between the different AD stages. Additionally, we explored the regions, showing significant changes based on the CorrTF extracted features’ strength among different AD stages. First, the process was initialized by applying the preprocessing on rs-fMRI data samples to reduce noise and retain the essential information. Then, the automated anatomical labeling (AAL) atlas was employed to divide the brain into 116 regions, where the intensity time series was calculated, and the CorrTF features were extracted for each region. The proposed framework employed the SVM classifier in two different methodologies, hierarchical and flat multi-classification schemes, to differentiate between the different AD stages for early detection purposes. The ADNI rs-fMRI dataset, employed in this study, consists of 167, 102, 129, and 114 normal, early, late mild cognitive impairment (MCI), and AD subjects, respectively. The proposed schemes achieved an average accuracy of 98.2% and 95.5% for hierarchical and flat multi-classification tasks, respectively, calculated using ten folds cross-validation. Therefore, CorrTF is considered a promising biomarker for AD early-stage identification. Moreover, the significant changes in the strengths of CorrTF connections among the different AD stages can help us identify and explore the affected brain regions and their latent associations during the progression of AD.

## Introduction

Alzheimer’s disease (AD) is a type of neurodegenerative disease, considered a crucial public health problem due to the increase of AD patients worldwide [1, 2]. The symptoms and cognitive abilities of AD patients decline over time as a result of the progressive degeneration and death of nerve cells, which affects the quality of life as it causes memory loss, difficulty in thinking, learning, performing familiar tasks, and determining the place, and time [3–7]. AD cognitive declination begins several years before becoming noticeable [4]. However, with the progression of this disease, the symptoms become more deleterious, which obstruct the ability of patients to perform their daily activities.

In 2017, AD was the sixth leading cause of death in the US, as it accounted for 121,404 deaths [4]. For elderly patients with age ≥ 65 years, diagnosed with AD or other dementia, the estimated healthcare costs $305 billion in 2020 [8]. However, up to date, available pharmacologic treatments of AD cannot stop or prevent the nerve cell destruction caused by the disease progression [4]. Consequently, early detection of AD is essential in improving the patient’s quality of life and society at a large scale. Furthermore, the early detection of AD while localizing the affected regions will allow researchers to improve the available treatment plan, which may help stop or prevent AD progression and severity. The diagnosis of AD is currently based on the medical history of individuals and their families and the physical and neurological examinations [8]. Additionally, resting-state functional magnetic resonance imaging (rs-fMRI), measuring the subtle changes in brain functional connectivity (FC), has become one of the emerging AD biomarkers [9].

Traditional machine learning techniques have been widely used to investigate rs-fMRI for AD identification [10–13]. Zhang et al. [10] used graph theory to measure and analyze the relationship between different brain networks’ connectivity changes using rs-fMRI data. They aimed at discriminating between EMCI and LMCI by calculating Pearson’s correlation coefficients from functional brain network time series at three different frequency bands with ranges; 0.01–0.08 Hz; 0.027–0.08 Hz; and 5: 0.01–0.027 Hz. The authors reported an accuracy of 83.9% using a dataset formed of 33 EMCI and 29 LMCI. Yuhu et al. [11] proposed identifying AD patients from NCs based on the FCs of activated voxels extracted using independent component analysis (ICA) from fMRI data. T-test was employed to study the significance of the correlation coefficient, calculated between the different activated voxel pairs. Finally, the SVM classifier was used to differentiate between AD patients and NCs. The authors achieved an accuracy of 92.9% using a dataset formed of 67 AD patients and 76 NCs. Furthermore, SVM was combined with graph theory to discriminate between MCI, AD, and NCs, where SVM trained using graph extracted features from 264 regions based on Power Brain atlas, achieving an accuracy of 88.40% using a dataset formed of 89 MCI, 34 AD, and 45 NCs [12]. Later, the same team employed Graph theory and SVM, applied on 90 regions, extracted based Automatic Anatomical Labeling (AAL) atlas [13]. This algorithm achieved 100% accuracy using a dataset formed of 20 AD and 20 NCs.

The transfer function approach had been employed to study brain activity using rs-fMRI as a new biomarker describing the propagation of visual information transferred between the brain’s visual areas [14–16]. Zayed et al. [14] investigated the significance of the correlation transfer function (CorrTF) in classifying the optic neuritis (ON) patients and NCs in two different conditions; opened eyes focused on a fixed point and closed eyes, where the visual information propagation transfer function was employed. The preliminary findings of this study suggest the potential usage of CorrTF in the analysis of rs-fMRI data to measure the impact of ON disease on the inter-regional communication between the visual areas of the brain. It is worth noting that the CorrTF was also employed in several studies distinguishing between ON and NCs [15, 16]. Both ON and AD of neurodegenerative diseases occur when nerve cells in the brain or peripheral nervous system lose function over time and ultimately die. All neurodegenerative diseases affect different brain regions and their way of communication. However, we aim to discover how CorrTF can help us identify AD stages based on altered brain communications.

Several studies have used rs-fMRI analysis and Deep learning algorithms to analyze and diagnose the different brain disorders [5, 17–23]. Suk et al. [17] aimed to diagnose MCI patients using deep learning techniques and state-space modeling. The authors trained a deep autoencoder (DAE) with the preprocessed mean intensity time signals, calculated for 90 AAL atlas brain regions to extract the nonlinear relations in a hierarchical and unsupervised manner. The extracted features were then fed to Hidden Markov Model (HMM) to model the functional dynamics for both MCI and NC. The authors employed an in-house collected dataset and the ADNI dataset, where the accuracy of 81% and 72.6%, respectively, has been achieved. Ronghui et al. [5] calculated Pearson’s correlation for the mean-time series using 90 brain regions extracted based on the AAL atlas. The correlation coefficients have been used to train the DAE with a softmax classifier. The algorithm was validated in a dataset of 91 MCIs, and 79 NCs, achieving 86.5% accuracy. Furthermore, Qureshi et al. [18] utilized a 3D-CNNs model, trained using rs-fMRI features extracted using Independent Component Analysis (ICA) to estimate the AD severity. They divided the AD patients into two groups according to the disease severity, named mild and moderate. The dataset consists of 77 and 56 for mild and moderate subjects, respectively. Their framework achieved an accuracy of 92.3%. Ramzan et al. [19] combined the deep residual neural networks (RNN) with the transfer learning approach to classify six stages of AD. They trained the RNN network using rs-fMRI volumes concatenated to form 2D Image/ subject. Using dataset consisted of 25 NC, 25 significant memory concerns (SMC), 25 EMCI, 25 LMCI, 13 MCI, and 25 AD. The authors reported an overall average accuracy of 97.9%.

This study aims at investigating the potential of the CorrTF as a feature extraction module to identify the different stages of AD patients. Moreover, we are interested in extracting features that describe the inter-regional communication between the different brain areas. The proposed framework feds the hierarchical scheme extracted CorrTF features, calculated for 116 brain regions, to a hierarchical scheme to discriminate between NC, EMCI, LMCI, and AD subjects.

## Materials and methods

### Dataset

The dataset employed in this study was downloaded from the Alzheimer’s Disease Neuroimaging Initiative (ADNI) database. The ADNI was launched in 2003 as a public-private partnership led by principal investigator Michael W. Weiner, MD. ADNI dataset consists of PET, different MRI structural and functional datasets, genetic data, clinical and neuropsychological examinations. The primary goal of ADNI is to test whether these techniques can be combined to diagnose and assess the progression of MCI and early AD. All ADNI studies are conducted according to the Good Clinical Practice guidelines, the Declaration of Helsinki, and U.S. 21 CFR Part 50 (Protection of Human Subjects) and Part 56 (Institutional Review Boards). Written informed consent was obtained from all participants before protocol-specific procedures were performed. The Institutional Review Boards approved the ADNI protocol of all participating institutions; for up-to-date information, see www.adni-info.org.

In this study, 371 subjects, formed of 189 female and 182 male, were employed from ADNI, categorized into four groups; normal controls (NCs), Early MCI (EMCI), Late MCI (LMCI), and AD patients, as reported in Table 1. The rs-fMRI images were acquired using 3.0 Tesla Philips Achieva scanners, where Echo Planner Imaging (EPI) scan protocol with Echo Time (TE) = 30 ms, Repetition Time (TR) = 3000 ms, flip angle = 80°, pixel size = 3.3 × 3.3 mm, acquisition matrix size = 64 × 64, and slice thickness = 3.3 mm, with 48 slices and 140 volumes.

**Table 1:** Overview of rs-fMRI study groups.

Study group	No. of subjects	Range of age
NC	167	65–96
EMCI	102	57–83
LMCI	129	57–88
AD	114	58–89

NC: Normal Control, EMCI: Early Mild Cognitive Impairment, LMCI: Late MCI, AD: Alzheimer Disease.

A complete description of ADNI is available at http://adni.loni.usc.edu/. Moreover, the data access requests are to be sent to http://adni.loni.usc.edu/data-samples/access-data/.

### Methodology

#### Data preprocessing and brain network analysis

**Fig 1:**
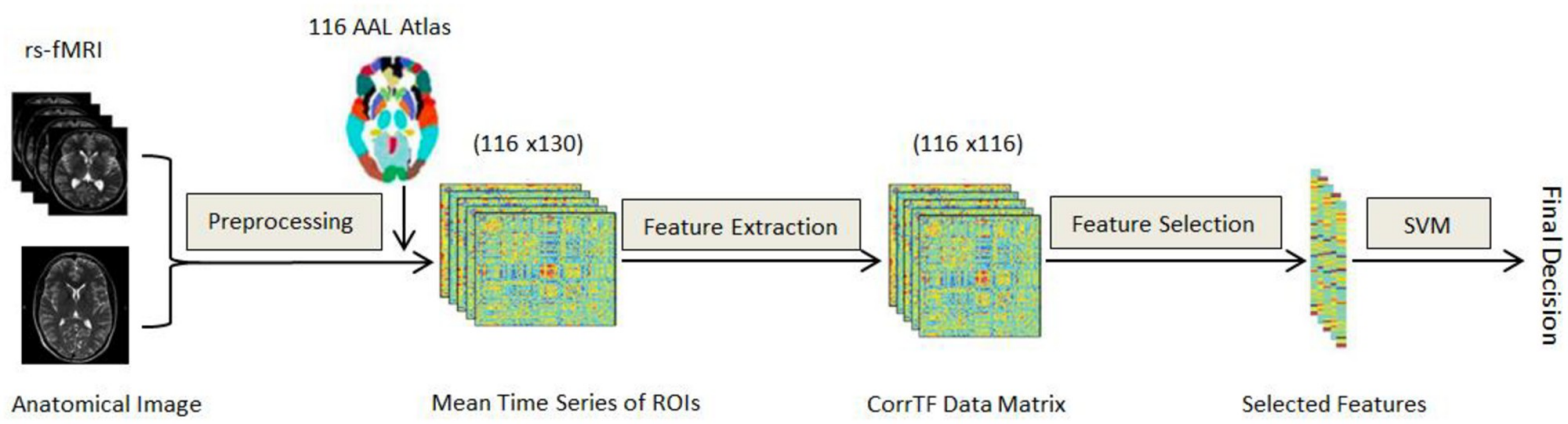
Block diagram of the AD diagnosing model using CorrTF features and CNN.

The standard preprocessing steps for rs-fMRI are performed using the Statistical Parametric Mapping (SPM12) software package [24], as shown in Fig 1. It includes discarding each subject’s first ten time-point volumes to guarantee magnetization equilibrium, slice time correction for interleaved acquisition, realignment for subject motion correction, and co-registration between the functional and structural images. The images were then normalized to the SPM12 EPI template and smoothed with a 4 mm full-width half-maximum Gaussian kernel. Later, each volume was divided into 116 regions of interest (ROIs) according to the AAL atlas, as listed in Table 2 [25–27]. Finally, the mean intensity time series for each ROI was extracted and filtered using a band-pass filter at 0.01–0.08 Hz to reduce non-neuronal contributions to blood-oxygenation-level-dependent (BOLD) signal fluctuations. Consequently, each subject was represented by a matrix with 116×130, representing the time-series signal for each region.

**Table 2:** The ROIs extracted from the different based on 116 AAL template.

ROI Label	Abb.	ROI Label	Abb.	ROI Label	Abb.
**Amygdala**	AMYG.	**Frontal_Mid_Orb**	ORBmid	**Precuneus**	PCUN
**Angular**	ANG.	**Frontal_Mid**	MFG	**Putamen**	PUT
**Calcarine**	CAL	**Frontal_Sup_Medial**	SFGmed	**Rectus**	REC
**Caudate**	CAU	**Frontal_Sup_Orb**	ORBsup	**Rolandic_Oper**	ROL
**Cerebelum_10**	CRBL10	**Frontal_Sup**	SFGdor	**Supp_Motor_Area**	SMA
**Cerebelum_3**	CRBL3	**Fusiform**	FFG	**SupraMarginal**	SMG
**Cerebelum_4_5**	CRBL45	**Heschl**	HES	**Temporal_Inf**	ITG
**Cerebelum_6**	CRBL6	**Hippocampus**	HIP	**Temporal_Mid**	MTG
**Cerebelum_7b**	CRBL7b	**Insula**	INS	**Temporal_Pole_Mid**	TPOmid
**Cerebelum_8**	CRBL8	**Lingual**	LING	**Temporal_Pole_Sup**	TPOsup
**Cerebelum_9**	CRBL9	**Occipital_Inf**	IOG	**Temporal_Sup**	STG
**Cerebelum_Crus1**	CRBLCrus1	**Occipital_Mid**	MOG	**Thalamus**	THA
**Cerebelum_Crus2**	CRBLCrus2	**Occipital_Sup**	SOG.	**Vermis_10**	Vermis10
**Cingulum_Ant**	ACG	**Olfactory**	OLF	**Vermis_1_2**	Vermis12
**Cingulum_Mid**	DCG	**Pallidum**	PAL	**Vermis_3**	Vermis3
**Cingulum_Post**	PCG	**ParaHippocampal**	PHG	**Vermis_4_5**	Vermis45
**Cuneus**	CUN	**Paracentral_Lobule**	PCL	**Vermis_6**	Vermis6
**Frontal_Inf_Oper**	IFGoperc	**Parietal_Inf**	IPL	**Vermis_7**	Vermis7
**Frontal_Inf_Orb**	ORBinf	**Parietal_Sup**	SPG	**Vermis_8**	Vermis8
**Frontal_Inf_Tri**	IFGtriang	**Postcentral**	PoCG	**Vermis_9**	Vermis9
**Frontal_Med_Orb**	ORBsupmed	**Precentral**	PreCG		

#### CorrTF feature extraction

The CorrTF measures the amount of information transferred from the input ROI to the output ROI. In this context, the characteristics of the functional connectivity path between any pairs of regions can be predicted. The destruction of the nerve cells during AD alters the connectivity path between the affected regions. Therefore, variations in the connectivity path might have the powerful ability to distinguish between normal and diseased subjects [15].

**Fig 2:**
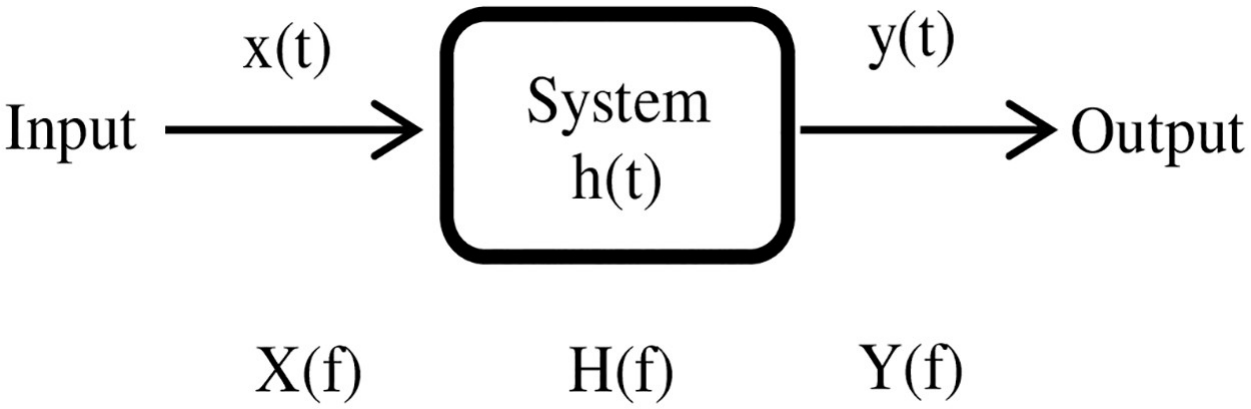
Representation of the transfer function H(f) for the system in time and frequency domain.

Theoretically, the transfer function models the system’s output for each possible input [28, 29], as shown in Fig 2. The relationship between output *y(t)* and input *x(t)*, for any system, can be modeled using

$$y(t)=\int_{-\infty }^{\infty }x\left(\tau \right)h(t-\tau )d\tau$$ (1)

where the *h(t)* is the impulse response that defines the system behavior. While the relationship in the frequency domain can be modeled using

$$Y\left(f\right)=X\left(f\right)H(f)$$ (2)

where *Y(f)*, *X(f)* and *H(f)* are the Fourier transform of the *y(t)*, *x(t*, and *h(t)* respectively. Similarly, this definition can be interpreted to model the connectivity path between any pair of brain regions, as illustrated in

$$CorrTF\left({ROI}_{1},{ROI}_{2}\right)=\left|\frac{F({ROI}_{1})}{F({ROI}_{2})}\right|$$ (3)

where *CorrTF (ROI*_*1*_, *ROI*_*2*_*)* is the transfer function calculated between the mean time series of *ROI*_*1*_ and *ROI*_*2*_, *Ƒ* is the discrete Fourier Transform. The correlation matrices were calculated using Eq (3) between each pair of the 116 ROIs [14]. The size of extracted CorrTF matrix would be 116×116×130 per subject. The CorrTF matrices were Fisher’s r-to-z transformed to standardize the feature vector to yield a zero mean and unit variance [30]. Each CorrTF matrix was averaged through the time points, which yielded the final feature matrix of 116×116. Among these features, we selected the discriminative features between normal subjects and AD different stages using a statistical t-test, where the selected features had a p-value less than 0.05.

#### Classification and performance evaluation

**Fig 3:**
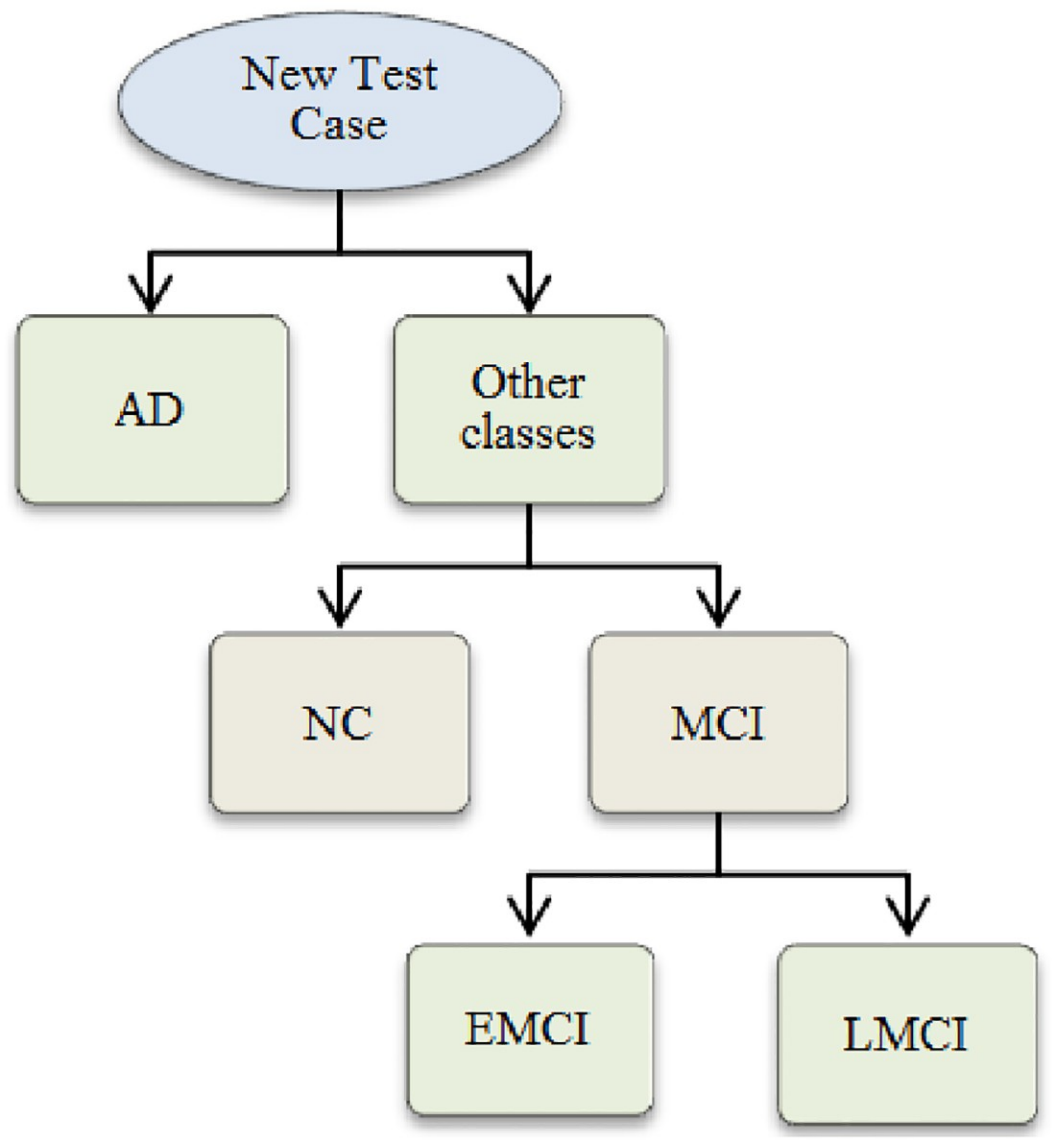
Hierarchical classification scheme to identify the stage of Alzheimer’s disease into NC, EMCI, LMCI, and AD.

Support vector machine (SVM) classifier was employed here to differentiate between the normal subjects and the different stages of AD. The SVM classifier is a supervised machine learning model that learns the hyperplane or a set of hyperplanes that gives the largest minimum distance to the training examples [31]. The SVM was trained using the significant CorrTF connections, selected using a statistical t-test. We adopt two classification schemes, named; flat and hierarchical multi-classification schemes. In the flat multi-classification scheme, a single machine was learned to classify any test case into four classes; NC, EMCI, LMCI, or AD. While, in the hierarchical scheme, Super-classes were formed by aggregating the subclasses to form binary classifiers following a one versus all concept [32], as shown in Fig 3. The hierarchical scheme usually improves the accuracy when the classes to be predicted are hieratically related [33]. Additionally, the learning of the different classifiers is independent of each other and can be implemented in parallel. Moreover, the hierarchical scheme helps to address some of the classes suffering from imbalanced dataset issues [34].

In this design, the mentioned hierarchy was selected based on the performance of the binary classification models’ accuracies, reported in Table 3, between each pair of classes, which minimizes the propagated error between the successive levels by classifying highly separated classes at top layers of the architecture. Furthermore, it takes advantage of the disease progression to differentiate between classes. In our hierarchical scheme, the first layer discriminates between the AD subjects and the rest of the classes. Then, the classifier initiates the second layer if the test case class is not AD. This layer’s classifier is to differentiate the incoming case into either NC or MCI. Finally, the third layer differentiates between the different classes of MCI, named EMCI or LMCI.

**Table 3:** Binary classification result accuracies (mean ± standard deviation).

Classes	Accuracy (%)
AD vs. NC	99.3 ± 1.5
AD vs. MCI	99.7 ± 0.9
NC vs. MCI	98.2 ± 1.7
EMCI vs. LMCI	1.00 ± 0

NC: Normal Control, EMCI: Early Mild Cognitive Impairment, LMCI: Late MCI, AD: Alzheimer Disease.

In order to evaluate the performance of the different schemes, a ten-fold cross-validation technique is employed to measure the accuracy, specificity, sensitivity, positive predictive value (PPV), and negative predictive value (NPV), calculated using the following equations:

$$Accuracy=\frac{TP+TN}{TP+FP+FN+TN}$$ (4)

$$Specificity=\frac{TN}{TN+FP}$$ (5)

$$Sensitivity=\frac{TP}{TP+FN}$$ (6)

$$PPV=\frac{TP}{TP+FP}$$ (7)

$$NPV=\frac{TN}{TN+FN}$$ (8)

Where TP: true positive, TN: true negative, FN: false negative, and FP: false positive.

## Results

This paper proposes investigating the CorrTF features’ effectiveness in distinguishing between AD’s three stages and normal subjects. The SVM classifier was trained hierarchically. Moreover, we compare the performance of the proposed hierarchical multi-classification scheme to the flat multi-classification scheme.

The model performance for hierarchical and flat multi-classification schemes is measured using five metrics; accuracy, sensitivity, specificity, PPV, and NPV, as reported in Table 4. Ten folds’ cross-validation was employed for testing to study the proposed system’s robustness. The average accuracy for both the hierarchical and flat multi-classifiers schemes is 98.2±1.4% and 95.5±4.3%, respectively, as listed in Table 4. Table 5 shows the confusion matrix for both the flat and the hierarchical multi-classifier. It can be observed that the hierarchical multi-classifier provided better discrimination of the NC from the rest of AD diseases that were misclassified in the case of flat multi-classifier. A comparison between the proposed model and similar studies employing the same dataset is found in Table 6.

**Table 4:** Classification performance of our model using CorrTF features (mean ± standard deviation).

Scheme	Accuracy (%)	Sensitivity (%)	Specificity (%)	PPV (%)	NPV (%)
Hierarchical Multi-Classifier	98.2 ± 1.4	99.4 ± 1.2	97.0 ± 3.1	98.6 ± 1.5	98.9 ± 2.4
Flat Multi-Classifier	95.5 ± 4.3	95.1 ±2.5	98.2 ± 4.0	98.6 ± 1.5	98.9 ± 2.4

PPV: Positive Predictive Value, NPV: Negative Predictive Value.

**Table 5:** Confusion matrix for: (a) Flat multi-classification, (b) Hierarchical multi-classification scheme.

(a)					
		**Ground Truth**			
		**NC**	**EMCI**	**LMCI**	**AD**
**Predicted**	**NC**	164	6	5	6
	**EMCI**	1	96	0	0
	**LMCI**	1	0	124	3
	**AD**	1	0	0	105
(b)					
		**Ground truth**			
		**NC**	**EMCI**	**LMCI**	**AD**
**Predicted**	**NC**	162	1	0	1
	**EMCI**	1	100	0	0
	**LMCI**	4	0	129	1
	**AD**	0	1	0	112

NC: Normal Control, EMCI: Early Mild Cognitive Impairment, LMCI: Late MCI, AD: Alzheimer Disease.

**Table 6:** Comparison with recently published work using ADNI dataset.

Method	Input Features	Classifier	Dataset	# Subjects	Accuracy (%)
**Khazaee et al.** [13]	Graph features	SVM	ADNI	20 NC, 20 AD	100
**Khazaee et al.** [12]	Graph features	SVM	ADNI	45 NC, 89 MCI, 34 AD	88.4
**Suk et al.** [17]	Mean-time series	DAE	ADNI	31 NC, 31 EMCI	72.6
			In-house collected dataset	25 NC, 12 MCI	81.1
**Ronghui et al.** [5]	Correlation matrix	DAE	ADNI	79 NC, 91 MCI	86.5
**Zhang et al.** [10]	Correlation matrix	SVM	ADNI	33 EMCI, 29 LMCI	83.8
**Ramzan et al.** [19]	Volumes concatenated to form 2D Image/ subject	RNN	ADNI	25 NC, 25 SMC, 25 EMCI, 25 LMCI, 13 MCI, and 25 AD	97.9
**Shi et al.** [11]	ICA	SVM	ADNI	76 NC, 67 AD	92.9
**Proposed Flat Scheme**	**CorrTF matrix**	**SVM**	**ADNI**	**167 NC, 102 EMCI**	**96.1**
				**129 LMCI, 114 AD**	
**Proposed Hierarchical Scheme**	**CorrTF matrix**	**SVM**	**ADNI**	**167 NC, 102 EMCI**	**98.2**
				**129 LMCI, 114 AD**	

NC: Normal Control, EMCI: Early Mild Cognitive Impairment, LMCI: Late MCI, AD: Alzheimer Disease, SMC: Significant Memory Concern, SVM: Support Vector Machine, DAE: Deep Autoencoder, RNN: Recurrent Neural Network, ICA: Independent Component Analysis.

**Fig 4:**
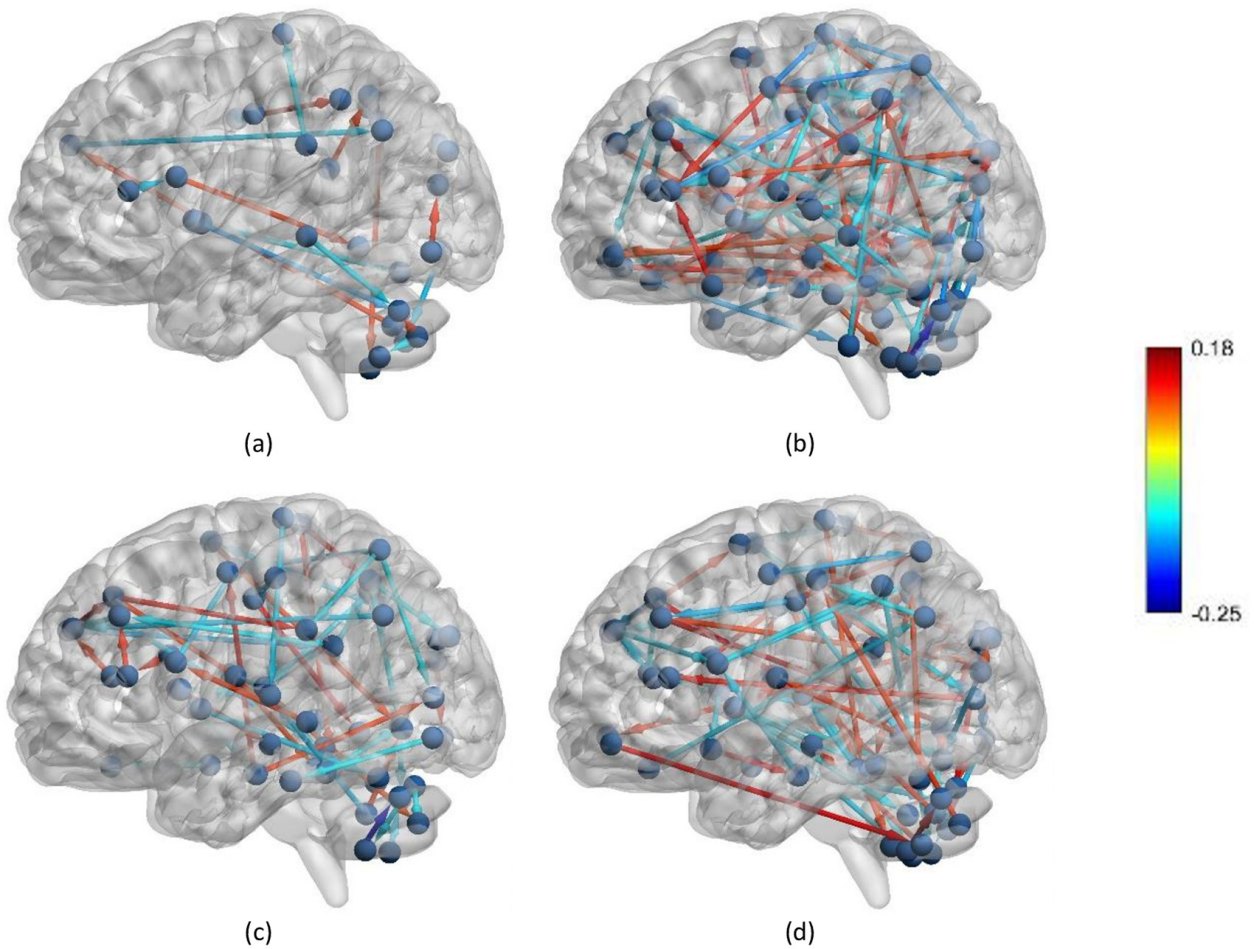
Brain connectivity networks for (a) CN, (b) EMCI, (c) LMCI, and (d) AD at threshold = 0.1, the color code defines the connection’s strength.

**Fig 5:**
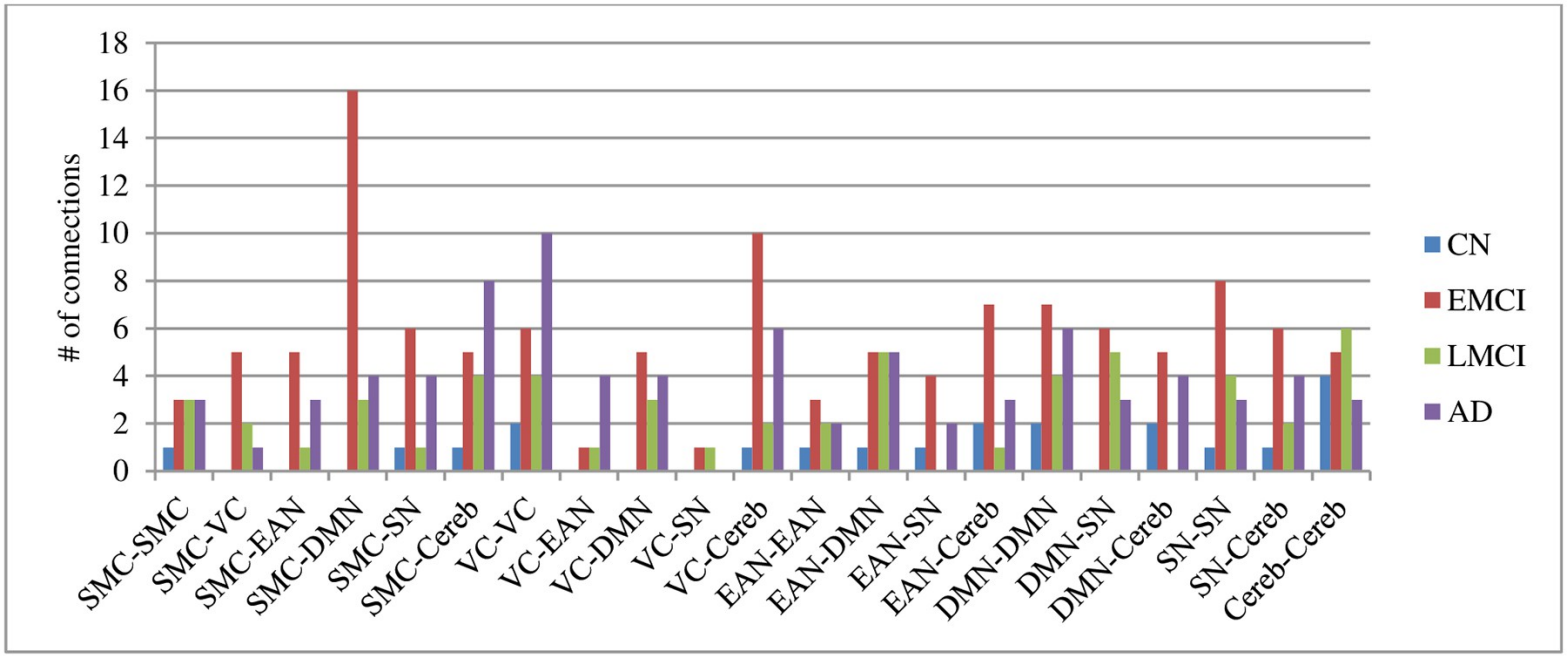
Number of connections with strength >0.1 grouped by input-output networks with connection’s directionality ignored. Sensorimotor Cortex (SMC), Visual Cortex (VC), Executive Attention Network (EAN), Default-Mode Network (DMN), Subcortical Nuclei (SN), and Cerebellum (Cereb).

The standard t-test was employed to investigate the CorrTF connections signal strength to extract the inter-regional communication between different brain areas among different groups. BrainNet Viewer software package [35] was employed to generate the topology of the brain networks after removing the connections with a strength less than 0.1 for the different subjects, as shown in Fig 4. According to Fig 4, the number of connections with high strength is increased in EMCI, an early stage of AD. Later, with disease progression, the number of connections with high strength started to decrease in LMCI, which started to increase once again in the case of AD. In Fig 5, the number of significant connections between each pair of networks for all AD stages was calculated and plotted for better visualization purposes. It is worth noting that within this analysis, the directionality of the connection has been ignored. As observed from Fig 5, the strength of connections between the following pair of networks, such as; (sensorimotor cortex (SMC)—visual cortex (VC)), (SMC—executive attention network (EAN)), (SMC—default mode network (DMN)), (VC–cerebellum (Cereb)), (EAN—subcortical nuclei (SN)), (EAN- Cereb), (SN–SN), and (SN–Cereb) were increased in case of EMCI while decreased during the progression of AD by at least two connections.

**Fig 6:**
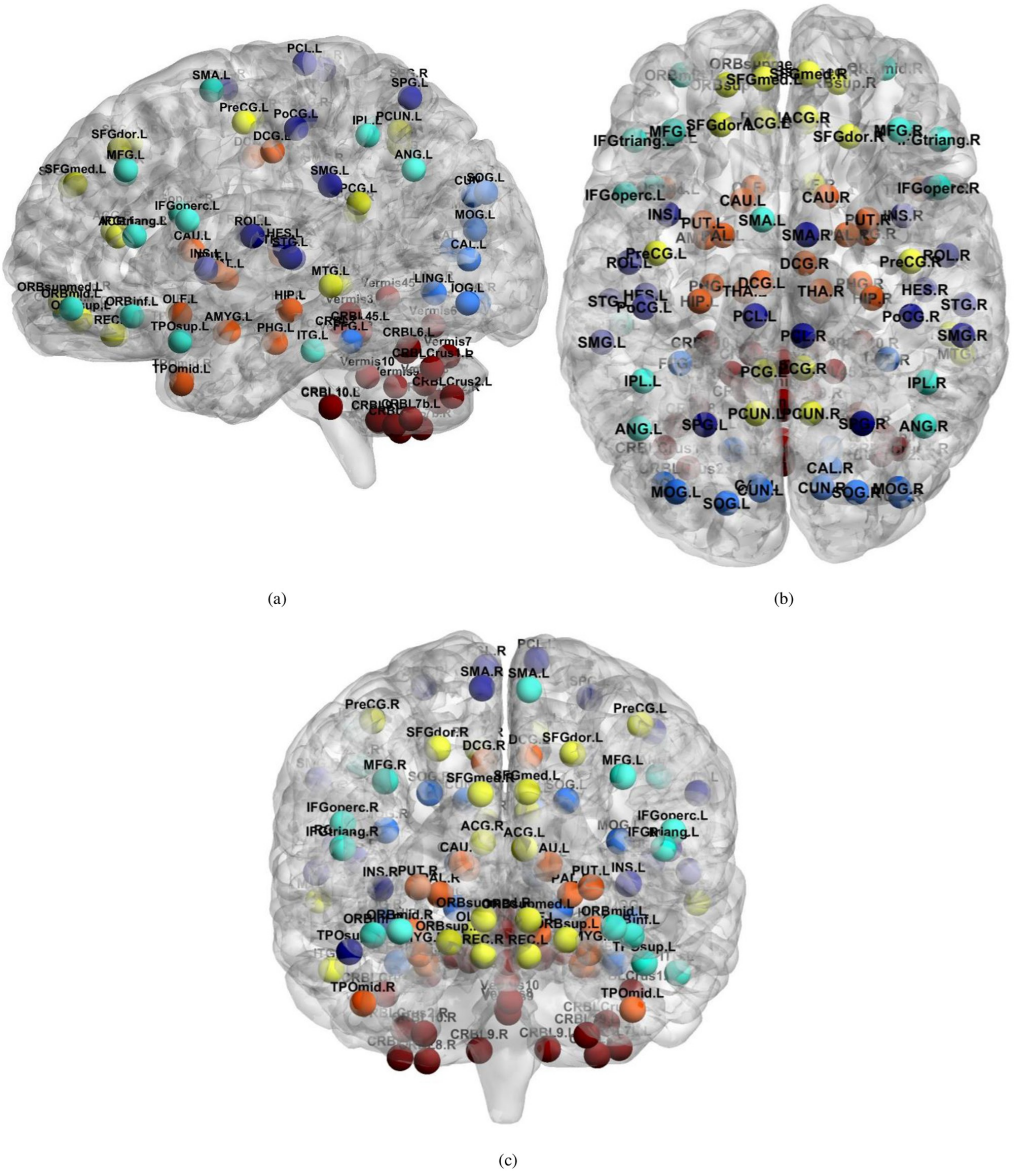
Localization of brain regions that have significant change in connections’ strength in case of AD compared to NC subjects AD, (a) Sagittal view, (b) Axial view, and (d) Coronal view. yellow: default-mode network; orange: regions of the subcortical nuclei; dark blue: regions of the sensorimotor cortex; blue: regions of the visual cortex; cyan: regions involved in the executive attention network; red: regions in the cerebellum.

On the other hand, the connections between the following pairs; (SMC–Cereb), (VC–VC), and (VC–EAN) were increased during the late stage of AD by at least two connections. Fig 6 shows the brain regions that have a significant change in connections’ strength in the case of AD compared to NC subjects. Among the brain regions in Fig 6, the highest contributions are for the cerebellum, DMN, and SMC areas. It is worth noting that the BrainNet Viewer software package [35] was employed to generate the topology of the brain networks for Fig 6. The most twenty discriminate connections are listed in Table 7, ordered according to their significance level.

**Table 7:** The most discriminative connections between CN and AD.

AAL ROI1	AAL ROI2	P-value
’Insula_L’	’Cerebelum_Crus1_R’	0.000031
’Parietal_Sup_R’	’Precuneus_L’	0.000183
’Rectus_R’	’Temporal_Sup_L’	0.000228
’SupraMarginal_R’	’Precuneus_R’	0.000229
’Cuneus_R’	’Thalamus_R’	0.000369
’Vermis_8’	’Frontal_Sup_Medial_R’	0.000439
’Frontal_Sup_L’	’Temporal_Sup_R’	0.000531
’Calcarine_L’	’Vermis_4_5’	0.000896
’Postcentral_L’	’Cerebelum_Crus1_R’	0.001035
’Frontal_Mid_Orb_L’	’Cerebelum_7b_L’	0.001109
’Frontal_Mid_Orb_L’	’Temporal_Mid_R’	0.001143
’Amygdala_L’	’Occipital_Inf_L’	0.001154
’Vermis_8’	’Heschl_R’	0.001171
’Cerebelum_Crus2_R’	’Cingulum_Mid_R’	0.001207
’Occipital_Inf_R’	’Cingulum_Ant_R’	0.001518
’Vermis_3’	’Insula_L’	0.001528
’Frontal_Med_Orb_L’	’Parietal_Inf_R’	0.001545
’Pallidum_L’	’SupraMarginal_L’	0.001572
’Precentral_R’	’Cuneus_R’	0.001865
’Angular_L’	’Cerebelum_3_R’	0.001873

AAL: Automatic Anatomical Labeling Atlas, ROI: Region Of Interest.

## Discussion

### Classification performance interpretation

In this study, we propose a hierarchical multi-classification scheme to distinguish between the different stages of AD. First, the system is initialized by the preprocessing module, which aims to reduce noise, volume normalization, and retain essential information. The preprocessed volumes were then segmented into 116 ROIs using AAL atlases. Then, the mean time series was calculated, followed by the CorrTF feature matrix extraction for each ROI. T-test was later employed to select the statistically significant features from the mean CorrTF feature set.

The performance metrics listed in Table 4 reports the accuracy, sensitivity, specificity, PPV, and NPV for both hierarchical and flat multi-classification schemes. The performance accuracy using the hierarchical multi-classification scheme has been boosted by 2.7% compared to the flat multi-classifier scheme. These results inform that breaking up the flat multi-classification problem into binary prediction steps can improve the classification performance without extracting extra features to obtain higher classification metrics.

Moreover, when the classes, to be predicted, are hierarchically related, the hierarchical multi-classification performance outperforms the flat multi-classification scheme [33]. Additionally, the hierarchical multi-classification scheme resolved the ambiguity in discriminating the NC from the different stages of AD. The proposed hierarchical multi-classification scheme was selected based on the performance of the binary SVM classifier for each pair of classes, as reported in Table 3. The PPV and the NPV metrics were employed to estimate the system classification prevalence as a diagnostic tool. In this context, among those who had a positive screening test, the probability of disease was 98.6%. While, among those who had a negative screening test, the probability of being disease-free was 98.9%. These give a high confidence level in the proposed model’s test results.

Since the model performance may rely on the training data, which may cause overfitting, 10-fold cross-validation was employed to prove the robustness since the standard deviation of the calculated performance accuracy is considered small. Comparing the flat multi-classification scheme to the hierarchical multi-classification scheme, the standard deviation for all performance measures has decreased in the case of the hierarchical multi-classification scheme, as reported in Table 4.

Table 6 compares the performance of the hierarchical multi-classification scheme to the recent research studies distinguishing the different AD stages. We can observe that employing the CorrTF in the flat multi-classification scheme boosted the performance compared to the models using other feature sets and regular machine learning tools described in [10–12]. Therefore, the extracted CorrTF features can find the hidden features that can discriminate between normal subjects and three stages of AD. Additionally, the employment of the hierarchical scheme even boosted the performance compared to that employing the deep learning algorithms [5, 17, 19], which is considered a less expensive tool in both learning and testing phases. Finally, it is worth noting that the dataset employed in this study is considered significant compared to the other studies, as listed in Table 6.

### Statistical analysis of CorrTF weights

Fig 4 shows the topology of the brain networks after removing connections with strength less than 0.1 for NC, EMCI, LMCI, and AD subjects. According to Fig 4, at an early stage of MCI number of connections with high strength is large compared to that in the case of the late MCI stage, while others increased during the late stage of AD. This means that the variations in the brain networks’ connections during the AD progression are non-monotonic [36]. It occurred due to the connection rearrangement occurring in the brain through the reparation of the neuro-functional losses caused by the disease progression [37]. Neuroplasticity, the ability of the brain to rebuild itself to form new neural connections to compensate for disease and injury and regulate their activities with new situations, may explain this phenomenon from the clinical aspect [38, 39]. Several researchers studied neuroplasticity and its effect on the different brain regions and their activities [39–42]. Clément et al. [40] found that the executive functions at EMCI benefit from neural reorganization, which breakdown during the late stages of the MCI, which was supported by our findings shown in Fig 5. In the case of EMCI, the connections between the EAN and cerebellum, SN and SMC ROIs have been increased in strength compared to NC subjects while decreased during the late AD stages. Kim et al. [36] reported that the brain network reorganization occurred stage-specific in a non-monotonic manner. Additionally, we can observe, from Fig 5, the high activation in SMC ROIs during the early stage of AD as coinciding with Wang et al. [43], Ferreri et al. [44], and Salustri et al. [45]. Furthermore, we can observe the increase in connections’ strength within the SN and between the SN and both DMN and EAN, as shown in Fig 5. This finding is supported by Mufson et al. [42], who stated that the hippocampus is capable of neural plasticity during MCI.

Fig 6 shows the brain regions that have a significant change in connections’ strength in the case of AD compared to NC subjects. The highest contributions of the affected connections are found in the cerebellum, DMN, and SMC networks. This comports with the decay in the regulator of motor activity and the modulation of cognition and emotional activities since the cerebellum is responsible for such tasks [46]. Affected cerebellum regions such as Cerebellum Cruses and Vermis, shown in Fig 6, are supported by Suk et al. [17], Jie et al. [47], and Olivito et al. [48]. In the case of NC, The DMN shows a high level of activation during brain resting conditions while not being involved in any explicit mental task [49]. However, in the case of AD, the level of activation is significantly affected, as shown in Fig 6, which agrees with Das et al. [49], Lee et al. [50], and Wu et al. [51]. Moreover, several studies supported the significant change in the connections to/ from DMN ROIs due to AD progression such as Precuneus_L, Precuneus_R, Rectus_R, Frontal_Sup_Medial_R, Frontal_Sup_L, Frontal_Med_Orb_L, Temporal_Mid_R, Cingulum_Ant_R, and ’Precentral_R, reported in Table 7 [5, 10–13, 17, 18, 49, 52]. Furthermore, recent studies indicated that the sensorimotor cortex ROIsinclude Insula_L, Parietal_Sup_R, Temporal_Sup_L, Temporal_Sup_R, Postcentral_L, Heschl_R, and SupraMarginal_L are also affected in the early progression of AD, which is also listed in Table 7 [5, 11, 47, 53–56].

Table 7 also confirms that there are eight out of 20 significant connections are connecting (cerebellum—SMC) and (DMN—SMC) [57–59]. This finding supports the clinical association between motor and cognitive function decline during AD progression [60, 61]. Additionally, several areas are affected by the AD progression based on the significant changes in CorrTF connections. This finding is in agreement with the literature, such as of VC; named Cuneus_R, Calcarine_L, Occipital_Inf_L, and Occipital_Inf_R [13, 62], the EAN, named as; Frontal_Mid_Orb_L, Parietal_Inf_R, and Angular_L [5, 11, 13, 47, 63] and SN named Pallidum_L, Cingulum_Mid_R, Amygdala_L, and Thalamus_R [5, 11, 47, 64].

## Conclusion

This study successfully discriminated between the different AD stages and healthy subjects using the correlation transfer function calculated for rs-fMRI data. Support Vector Machine (SVM) was employed in both flat and hierarchical multi-classification schemes to perform the classification task. A considerably large sample downloaded from the ADNI dataset was employed in this study. The CorrTF based features, calculated for 116 regions based on AAL Atlas, succeeded in providing latent details of the connections between the different regions since it measures the amount of information transferred between the input and output ROIs. The proposed schemes achieved an accuracy of about 98.2% and 95.5% for the hierarchical and flat multi-classification schemes, respectively. Also, we proved that the hierarchical schemes improved the classification performance measured in terms of accuracy, sensitivity, specificity, PPV, and NPV, without employing extra features. Moreover, the hierarchical scheme successfully identified the subtypes of MCI, named EMCI and LMCI, with an accuracy of 98% and 100%, respectively.

Furthermore, a statistical t-test was employed to identify the high strength connections. However, This study proved that CorrTF is a promising technique for extracting essential biomarkers for AD identification. Consequently, corrTF connections can further investigate the specific contribution of the different regions through the AD progression.
